# Therapeutic Potential of Bee Venom and *Capsicum annum* Hydroethanolic Extract in Experimental Model of Rheumatoid Arthritis in Male Rats

**DOI:** 10.1155/mi/5550872

**Published:** 2026-05-28

**Authors:** Ahmed Hussain, Amira Sh. Soliman, Mohamed Abd-Elbaset, Areej A. Al-Khalf, Eman A. Ahmed, Abdelwahab Khalil, Osama M. Ahmed, Mohamed S. Abbas

**Affiliations:** ^1^ Natural Resources Department, Faculty of African Postgraduate Studies, Cairo University, Cairo, Egypt, cu.edu.eg; ^2^ Department of Pharmacology and Toxicology, Faculty of Pharmacy, El Salehya El Gadida University, El Sharqia, Egypt; ^3^ Biology Department, College of Science, Princes Nourah Bint Abdulrahman University, Riyadh, 11671, Saudi Arabia; ^4^ Physiology Division, Zoology Department, Faculty of Science, Beni-Suef University, Beni-Suef, 62521, Egypt, bsu.edu.eg; ^5^ Entomology Division, Zoology Department, Faculty of Science, Beni-Suef University, Beni-Suef, 62521, Egypt, bsu.edu.eg

**Keywords:** anti-inflammatory, antioxidant, bee venom, Capsicum annum, CFA, gene expressions, rheumatoid arthritis

## Abstract

**Background:**

Rheumatoid arthritis (RA) is a long‐term inflammatory disease linked to higher mortality, joint degeneration, and long‐term disability. This study evaluated the anti‐inflammatory, antioxidant, and immunomodulatory effects of *Capsicum annuum* (CAP) hydroethanolic extract and bee venom (BV) in Freund’s complete adjuvant (FCA)‐induced RA in rats.

**Methods:**

Forty‐eight rats were divided into six groups: normal control (NC), RA control, and RA groups orally treated with methotrexate (MTX) (0.25 mg/kg), CAP (100 mg/kg), BV (0.25 mg/kg), and CAP (100 mg/kg) + BV (0.25 mg/kg). RA was induced by intradermal injection of FCA (100 μL/rat) into a footpad of the right hind paw at two consecutive days, and treatments were administered for 3 weeks. The study assessed serum rheumatoid factor (RF), anticyclic citrullinated peptide (ACCP) antibodies (ACPAs), tumor necrosis factor‐alpha (TNF‐α), interleukin‐4 (IL‐4), oxidative stress markers such as lipid peroxidation, and antioxidant parameters including superoxide dismutase (SOD) and reduced glutathione (GSH). Additionally, mRNA expression of various genes (inducible nitric oxide synthase [*iNOS*], endothelial nitric oxide synthase [*eNOS*], interleukin‐1β [*IL-1β*], *TNF-α*, matrix metalloproteinase‐1 [*MMP-1*], matrix metalloproteinase‐3 [*MMP-3*], and glutathione reductase [*GR*]) were analyzed, alongside histopathological evaluation.

**Results:**

The data showed that CAP and/or BV significantly reduced serum inflammatory and oxidative stress biomarkers, enhanced the antioxidant defense system, and improved the histopathological changes of ankle joint. Gene expression analysis revealed downregulation of *iNOS*, *eNOS*, *IL-1β*, *TNF-α*, *MMP-1*, and *MMP-3* gene expression, with upregulation of the *GR* gene expression. The combined CAP + BV treatment demonstrated the most potent antiarthritic effect.

**Conclusion:**

CAP and BV exhibit promising potential for alleviating RA in rats, with their combined application showing the greatest efficacy. The antiarthritic effects may be mediated via suppression of oxidative stress and inflammation and enhancement of the antioxidant defense system in addition to the modulatory effects on *MMP-1* and *MMP-3* gene expression. Further clinical research is essential to evaluate their safety and effectiveness in human RA management.

## 1. Introduction

There are more than 100 types of arthritis, a type of joint disease that involves inflammation in one or more joints. The most prevalent type of chronic inflammatory arthritis is rheumatoid arthritis (RA) [1]. It is typified by a joint attack that causes inflammatory synovitis, which frequently progresses to articular cartilage degradation, synovial tissue infiltration and growth, and subsequent tissue damage [2]. It is linked to higher mortality as well as significant morbidity and disability. About 1% of people worldwide are women, who are three times more likely than men to have RA [3].

Despite the pathophysiological basis of RA remains unclear, immunological processes correlated with genetic factors are thought to be the foundation for RA development [4]. It is hypothesized that RA patients either trigger a series of immunological reactions by reacting to autoantigens, like citrullinated peptides, or to a foreign peptide, like a bacterial or viral peptide [5]. Joint injury begins at the synovial membrane and affects the majority of the articular tissues due to the intricate connections between several immune modulators (cytokines and effector cells) [6]. Activated inflammatory cells, such as macrophages, T‐cells, and B‐cells, react to antigens in the joint by activating or releasing degenerative enzymes of other cells, tumor necrosis factor‐α (TNF‐α), antibodies, and reactive oxygen species (ROS) [7]. The disease progresses as a result of these mediators’ eventual induction of joint deformity and periarticular tissue deterioration [8].

A well‐known animal model that mimics a number of the clinical signs and symptoms of human RA is adjuvant‐induced arthritis (AIA). It helps to test different treatment modalities and gives a better understanding of the pathogenesis and pathways involved in the development of arthritis. One of the most well‐known allogenic agents used to create the AIA rodent model is Freund’s complete adjuvant (FCA). Because the histology and immunology of the rat model of RA caused by FCA are comparable to those of human RA, it is appropriate to screen new medications for RA treatment [9–13].

The number of people with RA is growing despite advancements in treatment, and the disease’s progression is not slowed by currently available or conventional medications like methotrexate (MTX), gold, biological agents, nonsteroidal anti‐inflammatory drugs (NSAIDs), glucocorticoids (steroids), or disease‐modifying antirheumatic drugs (DMARDs) [1]. MTX, a drug of DMARDs category, is used to treat many inflammatory diseases, but its long‐term use at high doses frequently induces tissue toxicity (hepatotoxicity, nephrotoxicity, and intestinal toxicity) by suppressing antioxidant enzymes and increasing free radicals [14–16]. As a result, the use of these conventional drugs is restricted due to the incidence of multiple adverse effects [17]. Thus, there is a pressing need to concentrate on different and more potent natural ingredients that come from plants or animals.

These days, there is increasing interest regarding herbal medicines because they have shown encouraging outcomes in treating a variety of medical conditions. Since ancient times, chili peppers, or *Capsicum* spp. as they are properly known, they have been a staple of many cultures’ cuisines. The most widely consumed and economically significant pepper for the food industry is *Capsicum annuum* (CAP), despite the fact that five other species have been domesticated and cultivated [18]. Its antibacterial, antimicrobial, anti‐inflammatory, and antioxidant properties have led to its use as a basic and active ingredient in food, cosmetics, and medications [18]. Conventional treatments have used it as an immunomodulator, appetite stimulant, counterirritant, and antibacterial to treat a range of ailments, such as parasitic infections, coughs, sore throats, and toothaches [7]. In many papers accumulated on RA, CAP is helpful in evaluating the chronic pain linked to the disease and its associated consequences [2, 19]. Large amounts of bioactive compounds, including carotenoids and capsaicinoids, are present in it. These compounds have a range of biological effects, including analgesic and anti‐inflammatory qualities that are similar to those of RA [20, 21]. It has been reported that capsaicinoids, particularly capsaicin, have antioxidant activities [22, 23]. Therefore, there is an increasing interest in researching the therapeutic effects of CAP for the treatment of this illness.

*Apis mellifera* uses bee venom (BV) as a weapon to protect honey and other objects. BV acupuncture therapy (BVT) has been utilized extensively as a complementary medicine to treat chronic illnesses, including RA, in many different countries since ancient times [3, 24]. It includes varieties of unique peptides, such as phospholipase A2 (PLA2), adolapin, mast cell degranulating peptide, melittin (a major component of BV), and apamine. BV has been reported to exhibit significant antioxidant properties, primarily due to its components, like melittin and apamin, which combat oxidative stress [25, 26]. It has become more and more common to use BV to reduce inflammation and discomfort associated with arthritis [27]. It has been elucidated that BV may lessen the signs and slow the progression of autoimmune diseases such as multiple sclerosis, RA, and systemic lupus erythematosus, even if no study has shown the molecular mechanisms behind the antiarthritic effects of BV treatment [28].

Therefore, the aim of this research was to investigate the possible advantages of BV both by itself and in conjunction with the CAP hydroethanolic extract in mitigating RA and reducing oxidation, inflammation, and immunological abnormalities associated with AIA in rats. The effects of BV and/or CAP were also compared with a reference conventional drug, MTX.

## 2. Materials and Methods

### 2.1. Chemicals

FCA, tris‐HCl, and ethidium bromide (IB) were obtained from Sigma–Aldrich Co. (St. Louis, MO, USA). Other high‐quality analytical chemicals and reagents were employed.

### 2.2. Preparation of CAP Hydroethanolic Extract

A Beni‐Suef, Egypt, commercial market is where the CAP fruits were bought. Staff members of the taxonomy department at Beni‐Suef University’s Botany Department in Beni‐Suef, Egypt, recognized and verified the CAP fruits. After thoroughly cleaning them with fresh water multiple times to guarantee that all contamination was gone, the CAP fruits were allowed to air‐dry for 7 days in a shady spot. The dehydrated fruits were ground into a coarse powder and left to macerate for 3 days at room temperature in 70% aqueous ethanol. The suspensions were permitted to be agitated regularly to completely mix the powder with 70% ethanol. The 70% aqueous ethanol extract was then filtered through a Whatman filter paper. The filtrate was evaporated under vacuum using a rotatory evaporator to yield an aqueous ethanolic extract of CAP fruits according to various publications [29–31]. The yield of the hydroethanolic extracts of fruits was 5% of dry weight. The viscous green crude extract was obtained and kept at −20°C pending its use in treatment. For the characterization of CAP hydroethanolic extract, high‐performance liquid chromatography (HPLC) analysis was performed to identify its chemical components.

### 2.3. Analysis of CAP Hydroethanolic Extract by HPLC

HPLC analysis was performed using an Agilent 1260 system with detection at 280 nm. Separation was achieved on a C18 column using 1% acetic acid and acetonitrile (50:50, v/v) at a flow rate of 1.5 mL/min. The injection volume was 20 μL and the column temperature was maintained at 40°C. Capsaicinoids were identified by comparison with authentic standards, and their relative percentages were calculated based on peak area normalization according to the previously described method [32].

### 2.4. Collection of BV

BV was obtained from *A. mellifera* by stimulating the bees with electric current pulses using the same method described by Kokot et al. [33]. It was delivered from the Egyptian Association of Beekeepers, Egypt. All samples were stored in darkness until analysis at −20 °C. The BV solution for HPLC studies was prepared by diluting 1 mg/mL in deionized water. A 0.45 μm membrane filter was used to filter the solution before it was loaded onto the column [34]. For animal injections, BV was freshly diluted in sterile physiological saline immediately before administration.

### 2.5. HPLC Analysis of BV

HPLC analysis of BV was carried out using an Agilent 1260 system with UV detection at 220 nm and a C18 column. A gradient system of 0.4% phosphoric acid and acetonitrile was applied at a flow rate of 1.0 mL/min. The injection volume was 20 μL, and the column temperature was maintained at 25°C. Apamine, PLA2, and melittin were identified by comparison with reference standards, and their relative percentages were calculated based on peak area normalization [35].

### 2.6. Experimental Animals

Male Wistar rats (110–130 g) were purchased from VACSERA (Egyptian holding company for serum and vaccines) animal house, Helwan station for experimental animals, Cairo, Egypt, and were selected randomly. The experimental protocol for this work was carried out in accordance with animal testing standards and authorized (Number BSU/FS/2018/32) by the Research Ethics Committee of the Faculty of Sciences at Beni‐Suef University in Egypt. Rats were kept in polypropylene cages with a regulated climate (25 ± 5°C outside, 45%–55% relative humidity, and a 12‐h light/dark cycle). They were also given free access to regular diet pellets and unlimited tap water. Before the experiment started, the animals had to spend a week becoming used to the lab environment. During this period, they were kept under observation to exclude any intercurrent infection. Anesthesia was used to collect samples from each animal, and every attempt was made to reduce the pain.

### 2.7. AIA in Rat

RA in male Wistar rats was induced by injecting 100 μL FCA, emulsion of heat‐killed *Mycobacterium tuberculosis* (each 1 mL FCA containing 1 mg *Mycobacterium tuberculosis*) into a footpad of the right hind paw of rats intradermally at 2 days that follow one another [36, 37]. Body weight (BW) and right leg paw anteroposterior thickness were measured to check for arthritis.

### 2.8. Experimental Protocol

Forty‐eight rats were allocated randomly into four groups (eight animals each) as follows:

1. Group 1 (Normal control [NC]): Rats included in this group were healthy and were given the equivalent volume of 1% carboxymethyl cellulose (CMC) by per os every other day for 21 days.
2. Group 2 (RA group): Rats in this group were RA‐induced rats that were given the equivalent volume of 1% CMC by per os every other day for 21 days.
3. Group 3 (RA + MTX group): Rats in this group were RA‐induced rats, which were treated with MTX (0.25 mg/kg dissolved in 5 mL 1% CMC) according to Koyama et al. [38] by per os every day for 21 days.
4. Group 4 (RA + CAP group): Rats in this group were RA‐induced rats, which were treated with CAP hydroethanolic extract (100 mg/kg dissolved in 5 mL 1% CMC) according to Vijayalakshmi et al. [39] by per os every day for 21 days.
5. Group 5 (RA + BV group): Rats in this group were RA‐induced rats, which were treated with BV (0.25 mg/kg dissolved in 5 mL 1% CMC) subcutaneously according to Lee et al. [40] by per os every day for 21 days.
6. Group 6 (RA + CAP + BV group): Rats in this group were RA‐induced rats, which were treated with CAP (as group 4) and BV (as group 5).

### 2.9. Morphological Examination

The anteroposterior thickness of the right leg hind paw in mm was determined for every animal by a micrometer. The values were taken just when the experiment was over.

### 2.10. Blood Sampling Collection and Tissue Processing

By the end of the experimental period, blood from each rat was obtained from the jugular vein of each under diethyl ether inhalation anesthesia. For use in biochemical analysis, the sera were obtained and stored frozen at −20°C after being separated by centrifugation at 2500 x *g* for 15 min at 20°C.

Following the collection of blood, the anesthetized animals were euthanized by cervical decapitation; each rat’s whole right ankle was removed right away and thoroughly cleaned with isotonic ice‐cold saline. The ankles of the right hind legs from five rats in each were isolated and were then preserved in a formal fixative solution for histopathological analysis, while the ankles of right legs of the other three rats in each group were rapidly dissected out for RT‐PCR analysis.

### 2.11. Estimation of Rheumatoid Factor (RF), ACCP, TNF‐α, and Interleukin‐4 (IL‐4) Levels

Using an enzyme‐linked immunosorbent test (ELISA), the levels of serum RF, anticyclic citrullinated peptide (ACCP), TNF‐α, and IL‐4 were assessed in accordance with the manufacturer’s kit instructions (MyBioSource, Southern California, San Diego, USA).

### 2.12. Detection of Oxidative Stress and Antioxidant Parameters

#### 2.12.1. Estimation of Lipid Peroxidation (MDA Level)

The malondialdehyde (MDA) level was analyzed according to the method of Mihara and Uchiyama [41]. The MDA content in nmol/mL in serum was measured at 532 nm.

#### 2.12.2. Estimation of Reduced Glutathione (GSH) Content

The serum‐reduced GSH content in nmol/mL was calculated using the described method [42]. The resulting yellow color was measured spectrophotometrically at 412 nm within 5 min.

#### 2.12.3. Estimation of Superoxide Dismutase (SOD) Activity

SOD was determined in serum based on the procedure of Marklund and Marklund [43], where at 430 nm, pyrogallol undergoes rapid auto‐oxidation in an aqueous solution, producing a yellow color. One unit was defined as the amount of SOD needed to inhibit pyrogallol auto‐oxidation at pH 7.8 at 25°C by 50%. The activity of SOD was expressed as U/mL.

### 2.13. Histopathological Examination

After the sacrifice, the right ankles were dissected out and fixed for 48 h in 10% neutral‐buffered formalin (NBF) and then sent to the pathology department of the National Cancer Institute in Cairo, Egypt, for hematoxylin and eosin staining (H&E) and paraffin section preparation. The 10% formic acid solution was used to decalcify the ankle. Before the specimen was embedded in paraffin wax, a graded ethanol series of dehydration was conducted after complete decalcification. Sections 5 µm thick were stained with H&E. The tissue sections were deparaffinized, mounted on glass slides, and stained with H&E for histological investigation using an electric light microscope (LEICA DM 2500). This allowed for the identification of any alterations in the tissue [44].

The prepared H&E‐stained sections were examined to detect ankle histopathological scores for synovial inflammation (synovitis), synovial hypertrophy (pannus formation), and articular cartilage erosion and damage. For each histological lesion, four grades were characterized: 0 = no lesion, 1 = mild, 2 = moderate, and 3 = severe according to previous publications [13, 45–47]. A score of 12 was the maximum possible.

### 2.14. RT‐PCR Analysis

The ankle of right hind leg of each rat was placed in a prechilled mortar and pestle and pulverized under liquid nitrogen to a fine powder or debris. The powdered tissue was immediately transferred to TRIzol Reagent (Zymo Research, CA, USA) and homogenized further using a homogenizer (Glas‐Col, Terre Haute, USA) to ensure complete lysis to extract total RNA. The COSMO PCR Master MixTM Kit (Willowfort, Co., UK) was then used to reverse‐transcribe the extracted RNA into cDNA, following the manufacturer’s instructions. Inducible nitric oxide synthase (*iNOS*), endothelial nitric oxide synthase (*eNOS*), interleukin‐1β (*IL-1β*), *TNF-α*, matrix metalloproteinase‐1 (*MMP-1*), matrix metalloproteinase‐3 (*MMP-3*), and glutathione reductase (*GR*) were the specific primers used in RT‐PCR to determine the target gene’s expression levels (Table 1). The PCR results were analyzed on a 1% agarose gel with IB staining, and the picture was generated using the Bio‐Rad Gel Doc XR System for gel imaging. The gray scale value was computed by quantitating the relative level of expression using ImageJ software. Every sample and gene were normalized using the β‐actin gene, which is a housekeeping gene [54].

**Table 1:** Gene sequence of *iNOS*, *eNOS*, *IL-1β*, *TNF-α*, *MMP-1*, *MMP-3*, and housekeeping gene *β-ACTIN*.

Gene	Primer sequence	Reference
*iNOS*	Forward: 5′‐TAC GGA GCA GCA AAT CCA C‐3′ Reverse: 5′‐GAT CAAAGG ACT GCA GCC TG‐3′	[48]
*eNOS*	5′‐ATGGAACAGTATAAGGCAAACACC‐3′ 5′‐GTT TCT GGT CGA TGT CAT GAG CAA AGG‐3′	[48]
*IL-1β*	Forward: 5′‐GGCAGTGTCACTCATTGTGG‐3′ Reverse: 5′‐AGG TGCTTG GGTCCTCAT‐3′	[49]
*TNF-α*	Forward: 5‐GCTGAGGTTGGACGGATAAA‐3 Reverse: 5‐AAAATCCTGCCCTGTCACAC‐3	[50]
*MMP-1*	Forward: 5′‐CCGGCAGAATGTGGAAACAG‐3′ Reverse: 5′‐GCTGCATTTGCCTCAGCTTT‐3′	[51]
*MMP-3*	Forward: 5′‐TTTGGCCGTCTCTTCCATCC‐3′ Reverse: 5′‐GGAGGCCCAGAGTGTGAATG‐3′	[51]
*GR*	Forward: 5‐ATTGGACGGGACCCAAATTCTAA‐3 Reverse: 5‐AGACATCGCCCACGGCATA‐3	[52]
*β-ACTIN*	Forward: 5‐TCACCCTGAAGTACCCCATGGAG‐3 Reverse: TTGGCCTTGGGGTTCAGGGGG	[49, 53]

It is important to note that before the start of therapy, animals were randomly assigned to experimental groups using a computer‐generated randomization list in order to reduce selection and observation bias. Investigators who were blind to the treatment groups performed all outcome assessments, including Western blot analysis, ELISA, histological evaluations, and RT‐PCR assays. To guarantee an objective assessment of treatment effects, group identities were hidden using coded labels until data analysis was finished.

### 2.15. Statistical Analysis

Utilizing computer‐based statistical software, SPSS‐27 (IBM Corp., 2020) [55], statistical analysis was performed. The data is given as mean ± SE. 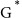
Power software was used to justify the adequacy of the sample size for detecting significant differences between groups. One‐way analysis of Variances (ANOVA) was used to statistically examine the data, and for multiple comparisons, a post hoc check for the least significant difference (LSD) was made. At a *p* <0.05, it was deemed statistically significant.

## 3. Results

### 3.1. HPLC Analysis of CAP Hydroethanolic Extract

**Figure 1:**
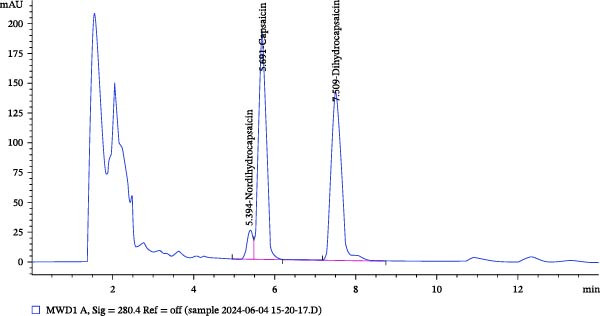
HPLC chromatogram of CAP hydroethanolic extract showing the presence of nordihydrocapsaicin, capsaicin, and dihydrocapsaicin.

The chromatogram obtained by HPLC for the CAP hydroethanolic extract at 280 nm (Figure 1) indicated the presence of nordihydrocapsaicin, capsaicin, and dihydrocapsaicin at relative percentages of 5.224%, 48.648%, and 46.127%, respectively.

### 3.2. HPLC Analysis of BV

**Figure 2:**
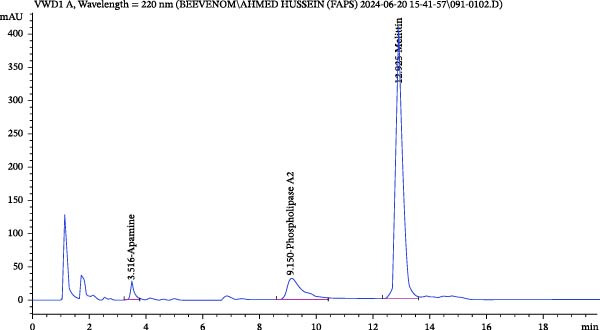
HPLC chromatogram of BV showing the presence of apamine, PLA2, and melittin.

The chromatogram obtained by HPLC for BV at 220 nm (Figure 2) indicated the presence of apamine, PLA2, and melittin at percentages of 2.863, 14.658, and 82.478% w/w of BV, respectively.

### 3.3. Effect on APTRHP and Serum RF, ACCP, TNF‐α, and IL‐4

The effect on the anteroposterior thickness of the right leg hind paw and serum RF, ACCP, TNF‐α, and IL‐4 levels in RA‐induced rats is shown in Table 2. The data exhibited a significant increase (*p* < 0.05) in anteroposterior thickness of the right leg hind paw and serum RF, ACCP, and TNF‐α levels accompanied by a substantial decrease (*p* < 0.05) in IL‐4 compared to their respective RA values. The MTX, CAP, BV, and CAP + BV, on the other hand, significantly produced a reduction in the elevated RFs and ACCP and proinflammatory cytokines (TNF‐α and IL‐1β) and substantially restored the anti‐inflammatory (IL‐4) level in arthritic rats to near normal values relative to the RA group at *p* < 0.05; the combined treatment produced the most efficient effect. Regarding the effects of treatments on the anteroposterior thickness of the right leg hind paw, BV alone and in combination with CAP is the most potent.

**Table 2:** Effects of MTX, CAP, BV, and CAP + BV on the anteroposterior thickness of right hind paw and serum RF, ACCP, *TNF-α*, and *IL-4* levels in FCA‐induced arthritic rats.

Parameters					
Groups	Right hind paw diameter (mm)	RF (mIU/mL)	ACCP (ng/mL)	*TNF-α* (pg/mL)	*IL-4* (pg/mL)
NC	5.05 ± 0.12^d^	19.3 ± 4.5^c^	1.5 ± 0.3^d^	32.8 ± 1.9^c^	84.1 ± 8.3^a^
RA	8.79 ± 0.31^a^	91.9 ± 7.4^a^	8.1 ± 1.5^a^	113.0 ± 8.1^a^	32.5 ± 2.6^c^
RA + MTX	7.23 ± 0.35^b^	38.8 ± 7.2^bc^	3.7 ± 0.1^bc^	49.7 ± 6.8^bc^	62.9 ± 8.9^ab^
RA + CAP	7.75 ± 0.19^bc^	51.8 ± 5.1^b^	4.0 ± 0.3^bc^	59.9 ± 7.5^b^	59.3 ± 6.8^b^
RA + BV	6.66 ± 0.29^c^	44.5 ± 8.0^bc^	5.0 ± 0.2^b^	57.3 ± 10.9^b^	53.4 ± 5.4^bc^
RA + CAP + BV	6.97 ± 0.42^bc^	29.2 ± 1.9^bc^	2.5 ± 0.3^cd^	41.1 ± 5.2^bc^	64.1 ± 8.5^ab^

*Note*: Data are expressed as mean ± SE. When two means in the same column do not share the same symbol(s) (a, b, c and d), they differ significantly at *p* < 0.05.

### 3.4. Effect on Oxidative Stress and Antioxidant Parameters

In the RA‐induced group, there was a significant elevation (*p* < 0.05) in MDA when compared with the respective NC group. On the other hand, MDA levels in arthritic animals treated with MTX, CAP, BV, and CAP + BV significantly exhibited a decrease (*p* < 0.05) compared to RA‐induced rats (Table 3). Concerning the reduced GSH level and the SOD activity, they exhibited a substantial decline (*p* < 0.05) in the RA group in comparison with the NC group. The MTX‐, CAP‐, BV‐, and CAP + BV‐treated arthritic groups significantly restored (*p* < 0.05) these lowered levels toward the normal levels (Table 3). CAP + BV was the most effective in improving the deteriorated MDA, GSH, and SOD levels.

**Table 3:** Effects of MTX, CAP, BV, and CAP + BV on serum MDA and GSH levels and SOD activity in FCA‐induced arthritic rats.

Parameter			
Groups	MDA (nmol/mL)	GSH (nmol/mL)	SOD (U/mL)
NC	0.32±0.02^a^	1.50 ± 0.06^a^	2.35 ± 0.09^a^
RA	1.38±0.03^e^	0.49 ± 0.04^c^	0.81 ± 0.04^e^
RA + MTX	0.63±0.04^c^	1.03 ± 0.04^b^	1.60 ± 0.06^c^
RA + CAP	0.81±0.02^d^	0.89 ± 0.01^b^	1.40 ± 0.06^d^
RA + BV	0.89±0.02^d^	0.94 ± 0.1^b^	1.35 ± 0.03^d^
RA + CAP + BV	0.39±0.02^b^	1.40 ± 0.06^a^	2.00 ± 0.06^b^

*Note:* Mean ± SE is used to express the data. When two means in the same column do not share the same symbols (a, b, c, d and e), they differ significantly at *p* < 0.05.

### 3.5. Effect on mRNA Expression of Ankle *iNOS, eNOS, IL-1β, TNF-α, MMP-1, MMP-3*, and *GR*

The current results as displayed in Table 4, depicted that the *iNOS*, *eNOS*, *IL-1β*, *TNF-α*, *MMP-1*, and *MMP-3* gene expressions in the RA group were substabtially (*p* < 0.05) raised in comparison with the NC group. However, the treatment of arthritic rats with MTX, CAP, BV, and CAP + BV induced a significant downregulation (*p* < 0.05) of the *iNOS*, *TNF- α*, *MMP-1*, and *MMP-3* gene expressions in the joint tissue relative to the RA. The treatments with MTX and CAP induced a significant decrease (*p* < 0.05) in *eNOS* mRNA expression, while BV and CAP + BV did not. All treatments except for BV produced a significant decrease in *IL-1β* mRNA expressions.

**Table 4:** Effect of MTX, CAP, BV, and CAP + BV on the mRNA expression levels of *eNOS, iNOS, IL-1β, TNF-α, MMP-1, MMP-3*, and *GR* in FCA‐induced arthritic rats.

mRNA level							
Groups	*eNOS*	*iNOS*	*IL-1β*	*TNF-α*	*MMP-1*	*MMP-3*	*GR*
NC	0.64±0.01^d^	0.59±0.00^f^	0.51±0.01^d^	0.50±0.01^f^	0.48±0.02^c^	0.46±0.01^c^	1.07±0.00^a^
RA	0.95±0.01^a^	1.06±0.03^a^	0.91±0.02^a^	0.99±0.01^a^	0.94±0.00^a^	0.93±0.00^a^	0.83±0.00^d^
RA + MTX	0.72±0.00^c^	0.65±0.01^e^	0.83±0.01^b^	0.73±0.01^d^	0.85±0.01^b^	0.88±0.01^b^	0.67±0.01^e^
RA + CAP	0.80±0.01^b^	0.78±0.01^d^	0.74±0.01^c^	0.65±0.01^e^	0.87±0.01^b^	0.89±0.01^b^	0.91±0.01^c^
RA + BV	0.93±0.01^a^	0.92±0.01^b^	0.91±0.02^a^	0.83±0.01^b^	0.87±0.01^b^	0.89±0.01^b^	0.85±0.01^d^
RA + CAP + BV	0.94±0.00^a^	0.83±0.01^c^	0.84±0.01^b^	0.78±0.00^c^	0.85±0.00^b^	0.88±0.01^b^	0.95±0.00^b^

*Note*: Mean ± SE is used to express the data. The number of tested samples in each group is three. When two means in the same column do not share the same symbol(s) (a, b, c, d, e, and f), they differ significantly at *p* < 0.05.

Furthermore, the results of the study also explored a substantial (*p* < 0.05) downregulation of the *GR* gene expression in the RA group concerning the NC group. On the other hand, treatment with CAP and combined CAP + BV resulted in a substantial (*p* < 0.05) upregulation of the *GR* gene expression relative to the RA group, but its expression was still downregulated with MTX‐ and BV‐treated arthritic groups.

### 3.6. Effect on Histopathological Examination of Joint

**Figure 3:**
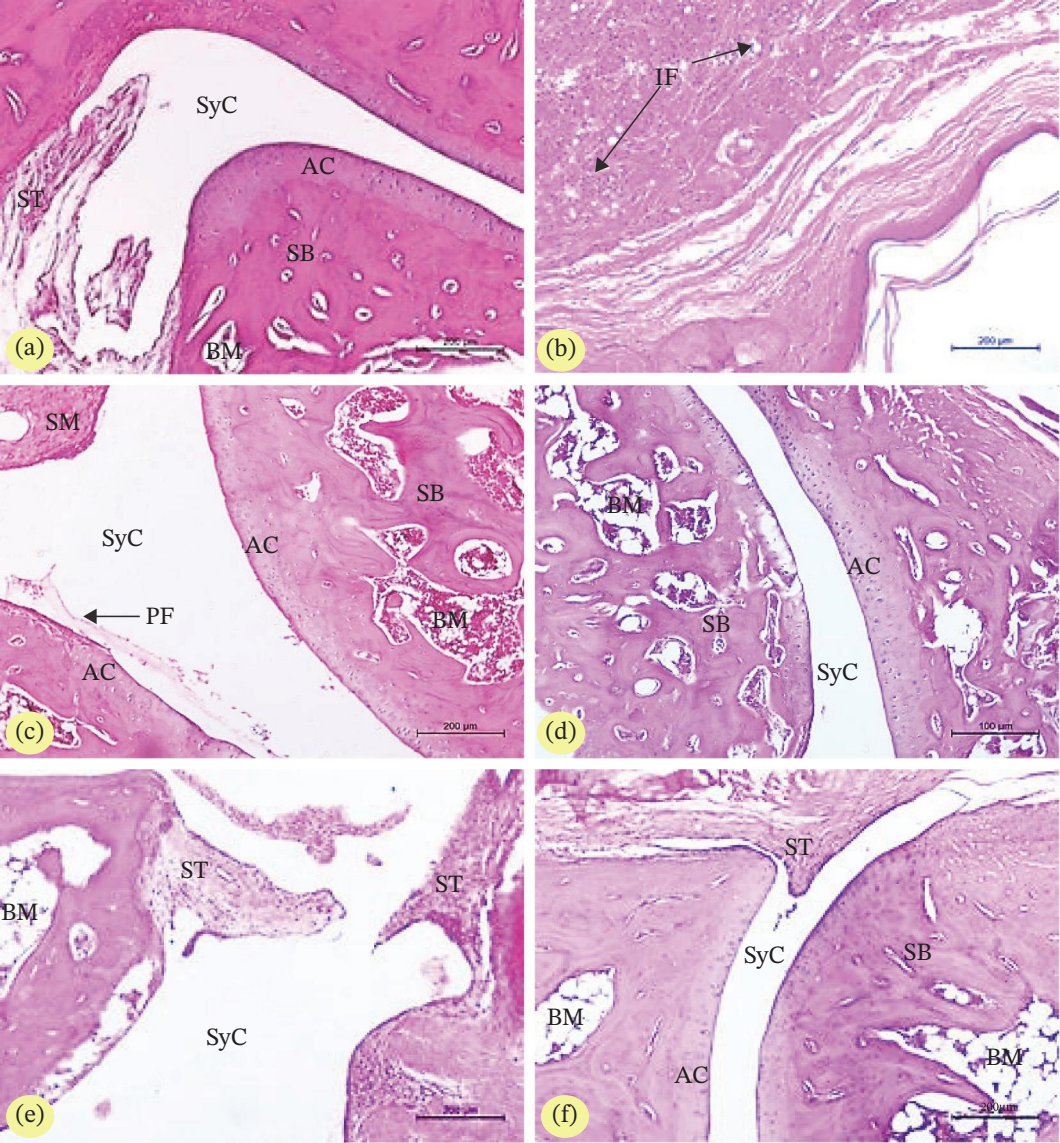
Photomicrographs of the section in ankle joints of normal (a), arthritic control (b), and arthritic rats treated with MTX (c), CAP (d), BV (e), and CAP + BV (f). Photo (a) depicted normal articulating cartilage (AC), synovial tissue (ST), synovial cavity (SyC), sponge bone (SB), and bone marrow (BM). Photo (b) showed deep subcutaneous inflammatory cells infiltration (IF). Photo (c) showed nearly normal articular cartilage and subchondral bone associated with hyperplastic synovial membrane. Photo (d) showed normal articular cartilage and subchondral bone. Photo (e) depicted proliferation at the synovial membrane and tearing of the synovial capsule with moderate inflammation of synovial tissue (ST). Photo (f) showed mild hyperplasia in the synovial tissue (ST) with a normal articular surface. Scale bar 200 μm refers to 100x magnification and scale bar 100 μm refers to 200x magnification.

As shown in Figure 3, histopathological analyses support the results of serum enzymes and RA biomarker assays. In NC rats, no associated pathological indications of arthritis were found. The synovium, articular cartilage, and subchondral bone exhibited normal histological structures (Figure 3a). In contrast, pathological sections of RA group rats showed that the arthritis tissues had obvious pathological changes, including extremely severe subcutaneous granulomatous inflammatory reactions (Figure 3b). Photomicrographs (c, d, and f) indicated that MTX, CAP, and CAP + BV markedly improved the inflammatory response and reduced cartilage and bone destruction, inflammatory cell infiltration, cartilage surface erosion, and joint degeneration when compared to the RA model group. However, the RA group treated with BV exhibited thickening of the synovial membrane and tearing of the synovial capsule (Figure 3e).

**Figure 4:**
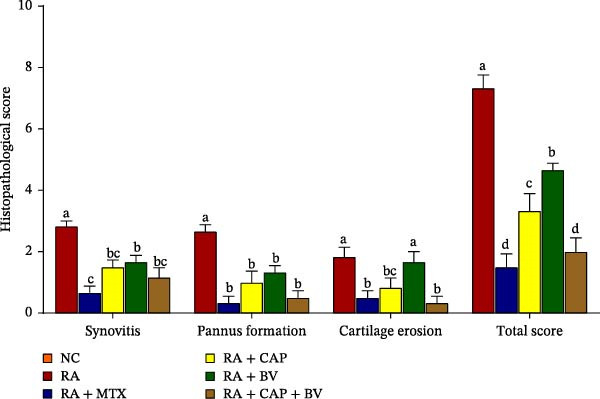
Effect of MTX, CAP, BV, and CAP + BV on ankle histopathological scores in FCA‐induced arthritic rats. When two means for the same histopathological score do not share the same symbol(s) (a, b, c and d), they differ significantly at *p* < 0.05. Score 0: no lesion, 1: mild, 3: moderate, and 3: severe.

H&E‐stained sections of the ankle joints from the right hind legs were examined for detection of synovial inflammation (synovitis), pannus formation, articular cartilage bone erosions, and total score, with each parameter scored on a scale from 0 to 3 (Figure 4). With the exception of the effects of BV on cartilage erosion, all histopathological scores exhibited a significant decrease (*p* < 0.05) as a result of treatments with MTX, CAP, BV, and CAP + BV. The combinatory effect of CAP and BV was more potent than the separate treatments with CAP and BV. Moreover, the effect of treatment with CAP + BV was comparable to the effect of MXT since the two groups have no significant difference (*p* > 0.05) when they were compared with each other.

## 4. Discussion

One percent of people worldwide suffer from RA, a common inflammatory disease [56]. In addition to causing joint injury and a reduction in quality of life, it also results in significant medical and social costs [57]. RA is more common in women than in men, and its prevalence increases with age [58]. RA is a complicated interplay between the environment and the host that influences the overall risk of susceptibility, persistence, and severity, even if the precise etiology has not yet been determined [59]. The production of chemokines and cytokines that boost the immune response and contribute to chronic inflammation and bone loss also occurs as a result of the immunological complexes in RA joints drawing immune cells by complement or direct cell activation [60].

There are still unmet needs despite the recent emergence of many effective immune‐targeted drugs that have greatly improved the prognosis of the disease. Despite encouraging experimental research, the exploration of clinical diagnoses, and therapeutic management techniques, there is currently no comprehensive treatment for all bone and cartilage issues [61]. The use of DMARDs with different amounts of short‐term glucocorticoids has been the primary focus for the last 20 years. According to data, those with RA have a higher risk of infections and cancers like lymphoma and lung cancer than those without the condition. In particular, the use of conventional synthetic DMARDs (csDMARDs) and biologic (b)/targeted synthetic (ts) DMARDs raises the risk of infection in RA patients [62]. MTX, a member of the csDMARD class, often causes significant adverse effects as a result of long‐term treatments by creating an imbalance between ROS production and the body’s antioxidant defense system leading to chronic liver and kidney injuries [15, 16].

According to earlier reports, scientists’ ambition to assist those patients in managing their illness and, as a result, overcoming their sufferings without or with the least amount of difficulties is always expanding. As a result, many researchers are worried and very interested in how well natural remedies work to heal diseases of the bones and cartilage. In contrast to synthetic immune‐suppressing medications and their detrimental effects on RA patients, our study demonstrated the effectiveness and utility of two natural agents, such as BV and/or CAP, against FCA‐induced arthritis. Additionally, the number of manifestations and biomarkers was tracked in order to examine the effectiveness of these medications.

Synovitis, or inflammatory expansion of the synovial membrane, is a hallmark of RA disease and can cause swelling in the joints [63]. Therefore, the thickness of the right hind paw was measured in order to determine and quantify paw edema, which was thought to be an indicator of edema and the onset and progression of RA. To estimate the anti‐inflammatory effect of the additional treatments, the thickness of the rats’ right hind paws was measured at the end of the experiment and compared between the NC group and the arthritic‐treated groups. The RA group’s right hind paw anteroposterior thickness was significantly thicker than the NC group’s, according to our measurements. Given that the FCA model is characterized by severe paw edema, synovial development, and the loss of articular tissues, including bone and cartilage, this was dependable [36, 37]. However, the anteroposterior thickness of the right leg hind paw was considerably reduced by all applied treatments in the vicinity of the normal rats and BV, with CAP + BV therapies showing the greatest efficacy. Our findings are in line with those of the Hazekawa et al. [64] research team, who conducted an in vitro investigation on mouse spleen‐cultured cells and found that water extract from bell pepper leaves had an anti‐inflammatory effect that was mediated as an immunosuppressive reaction against T‐cell activation. They also demonstrated the potential mechanisms behind that effect [64]. Additionally, Qiao et al. [65] recently demonstrated that pepper extracts, at concentrations ranging from 0 to 100 g, produced a notable anti‐inflammatory effect and dose‐dependent reduction of oxidation. Our earlier research [66] shown the significant effectiveness of BV therapy in reducing edema and inflammatory tissues. The conventional BV injection into an acupuncture point also reduced arthritis‐related edema and nociceptive responses in rats with FCA‐induced arthritis, according to Lee et al. [67].

Autoantibodies that may be examined in the serum and synovial fluid (SF) may be present during a pre‐RA phase that lasts for months to years prior to the start of the disease phenotype. These include RF and ACCP, which are currently employed as diagnostic biomarkers [68].

An important turning point in the creation of immunological biomarkers for autoimmune diseases was the close correlation between RF and RA. Subsequently, the specificity of ACCP, which recognizes antibodies, is >95% for RA and approximately 86% for RF. Citrullinated peptides promote T‐helper (Th) cell proliferation and are linked to an increased risk of RA incidence by attaching to human leukocyte antigen‐DRB1 (HLA‐DRB1) molecules that share an epitope [69]. This association led to significant advancements in our understanding of the pathophysiology of RA in addition to providing a biomarker with greater specificity for RA (approximately two‐thirds of RA) [70]. Van Delft and Huizinga [71] claim that they might form immunological complexes in the joints that draw in immune cells by complement or direct immune cell activation, which causes chemokines and cytokines to be released. These behaviors have the potential to increase bone loss, chronic inflammation, and the immunological response.

Accordingly, the current data demonstrated that the incidence of the disease and associated symptoms were demonstrated by the significantly greater concentration of both FR and ACCP autoantibodies in the RA‐arthritic rats as compared to the normal group. Conversely, the combinatory CAP + BV group was the most prevalent, and all of the studied medications significantly decreased these associated antibodies. These results suggest that CAP and BV, either separately or in combination, have therapeutic and protective effects in comparison to the usual medication, MTX. Their combined ability to selectively modulate immune responses, limit inflammatory reactions, including the development of autoantibodies that establish the disease, and explain their antiarthritic capabilities may be the reason for these outcomes. The synergistic effects of CAP and BV may be the reason for the most powerful efficacy of CAP + BV.

The chronic infiltration of effector T cells, B cells, and other innate effector cells into the joints causes persistent synovitis, which is one of the characteristics of RA. In order to aid in the breakdown of cartilage and bone, these immune cells build a complex network that stimulates the production of several cytokines, chemokines, adhesion molecules, and MMPs, which are enzymes that break down the extracellular matrix. Similar to macrophages, innate immune cells like mast cells and neutrophils increase the release of small‐molecule mediators of inflammation like ROS, nitrogen intermediates, and prostanoids, as well as proinflammatory cytokines like TNF‐α, IL‐1β, and IL‐6, which leads to the development of synovitis [36, 37].

**Figure 5:**
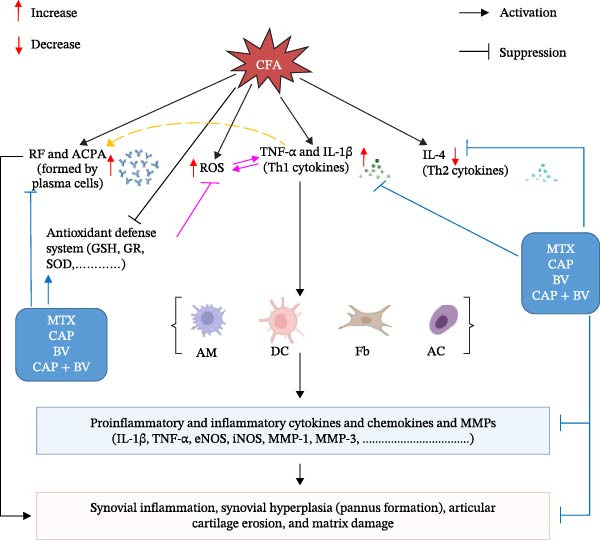
Schematic diagram showing the modes of action of MTX, CAP, BV, and CAP + BV treatments in FCA‐induced arthritic rats. AC, activated chondrocyte; ACPA, anticyclic citrullinated peptide antibody; AM, activated macrophage; DC, dendritic cell; eNOS, endothelial nitric oxide synthase; Fb, fibroblast; GR, glutathione reductase; GSH, glutathione (reduced form); IL‐1β, interleukin1‐beta; IL‐4, interleukin 4; iNOS: inducible nitric oxide synthase; MMP‐1, matrix metalloproteinase‐1; MMP‐3, matrix metalloproteinase‐3; RF, rheumatoid factor; ROS, reactive oxygen species; SOD, superoxide dismutase; Th1, T helper 1; Th2, T helper 2; TNF‐*α*, tumor necrosis factor‐alpha.

Long believed to be true, RA is caused by macrophages polarizing into a proinflammatory “M1” phenotype, which leads to the production of proinflammatory mediators and a reduction in the “M2” phenotype of regulatory and anti‐inflammatory cytokines, such as IL‐4, IL‐13, and IL‐10. The induction of RA is caused by the preponderance of proinflammatory cytokines over anti‐inflammatory cytokines [72]. Therefore, the novel therapeutic medicines that were explored to combat the disease focused on either blocking or modifying these M1 and Th1 phenotypic pathways. The determination of proinflammatory cytokine (TNF‐α) and anti‐inflammatory interleukin (IL‐4) levels in serum as well as ankle *TNF-α*, *IL-1β*, *iNOS*, *eNOS*, *MMP-1*, *MMP-3*, and *GR* gene mRNA expression levels within tissues was the main goal of this investigation. In our study, the treatments of arthritic rats with MTX, CAP, BV, and CAP + BV produced downregulation of Th1 cytokines and upregulation of anti‐inflammatory cytokine (IL‐4). Therefore, in our opinion, the treatments may influence Th and macrophage phenotypes, promoting Th1/M1‐like characteristics and favoring Th2/M2‐like responses (Figure 5).

Our data showed that the RA animals had a marked autoimmune inflammatory condition caused by arthritis induction, as evidenced by a considerable increase of the inflammatory *TNF-α*, *IL-1*β, *iNOS*, *eNOS*, *MMP-1*, and *MMP-3* expression levels compared to β‐actin. These results came after a study by Totoson et al. [73] that showed that in the AIA animal model, the modulation of immune mediators was more inflammatory than anti‐inflammatory. On the other hand, all of the mRNA expression of inflammatory mediators’ genes that were evaluated (*TNF-α*, *IL-1*β, *iNOS*, *eNOS*, *MMP-1*, and *MMP-3*) had downregulated levels in the data from the treated arthritic animals. This may be explained by *TNF-α*’s critical function in triggering inflammation and the development of arthritis (Figure 5). It encourages the expression of additional proinflammatory cytokines such as *IL-1*β, enhances the *iNOS* expression, and in turn induces the development of excessive levels of nitric oxide (NO) [74]. Similarly, *iNOS* promotes the expression of MMPs family (*MMP-3*, *MMP-13*, and *MMP-9*) increasing extracellular matrix breakdown and cartilage injury [75]. Therefore, the degree of inflammation is decreased whenever the expression of the TNF‐α pathway is particularly suppressed (Figure 5). These results are also in line with other oxidative stress and edema metrics that point to the studied treatments’ anti‐inflammatory properties. Melittin, the main component of BV, as determined by HPLC analysis in this study, may be the cause of the anti‐inflammatory benefits of BV. Previous studies have demonstrated melittin’s anti‐inflammatory properties [40, 76].

Redox coupling glutathione–glutathione disulfide (GSH/GSSG) and other extracellular and cellular redox buffering systems maintain redox homeostasis. GSH is the primary redox buffer in most cells because it is responsible for maintaining the environment’s general reduction state. It can scavenge ROS and then become oxidized, forming a disulfide bridge between two GSH molecules (oxidized dimer or GSSG); consequently, it maintains redox homeostasis. GR is an important enzyme that recycles the oxidized glutathione, GSSG, back into its reduced form, GSH [77]. The balance of these boosting systems is regulated by key antioxidant enzymes such as catalase, SOD, and the selenoproteins glutathione peroxidase and thioredoxin reductase, as well as nonenzymatic antioxidants such α‐tocopherol (vitamin E), ascorbate (vitamin C), β‐carotene, and flavonoids [78]. Any time there is an imbalance favoring oxidants over antioxidants, this redox homeostasis is disrupted [79].

However, excessive ROS generation brought on by over‐oxidation damages and modifies lipids, proteins, and DNA, among other cell components. As a result, lipids in cell or organelle membranes undergo peroxidation, which produces conjugated bioactive aldehydes like MDA or thiobarbituric acid reactive substances (TBARSs), which directly harm phospholipids and serve as indicators of cell death that trigger apoptosis or cell‐programed death [80]. According to Mukhopadhyay et al. [81], oxidative stress plays a critical role in the incidence of RA. Both locally and systemically, they found a considerable rise in ROS, and they also found a strong link between RA patients’ disease activity and increased ROS generation in peripheral blood neutrophils. They suggested somatic mutation, oxidative modification of autoantigens, changes in cell signaling, and anomalies in DNA damage repair pathways as possible causes of ROS‐mediated cell harm. The rise in ROS level can trigger inflammation, and it acts as a crucial, dual‐faceted mediator of inflammation, acting as a second messenger that triggers inflammatory signaling cascades as well as a direct damaging agent in RA (Figure 5). Prior publications provided support for these explanations [13, 47]. Ablation of the excessive production of ROS by antioxidants could have important roles in suppressing inflammation and ROS‐induced damage and deteriorations in RA.

Our findings were in complete agreement with these publications, and the RA group showed a marked rise in MDA, a consequence of peroxidation, along with a decrease in serum levels of the antioxidants GSH and SOD and *GR* expression in ankle tissues. Conversely, the treatment of arthritic groups with CAP and/or BV resulted in a notable decrease in MDA levels and oxidative damage, improved the antioxidant defense system, as evidenced by higher levels of GSH and SOD activity in the sera samples, and increased the amount of *GR* mRNA expression found in articular tissues. Out of all the treatments, the CAP + BV treatment was generally the most effective. According to the HPLC study, the phenolic components in CAP, such as capsaicin, dihydrocapsaicin, and nordihydrocapsaicin, may be responsible for its effectiveness. According to Sinisgalli et al. [82], these phenolic compounds have the ability to reduce and neutralize free radicals, which increases antioxidant activity and restores redox equilibrium. In our earlier investigation, BV therapy also demonstrated a strong antioxidant effect [66]. The LPO profile clearly decreased as a result of treatment, while the GSH content and SOD activity both markedly improved. The antioxidant efficacy of BV may be due to its components, like melittin and apamin, which combat oxidative stress [25, 26]. In general, a significant oxidant/antioxidant balance was indicated by the combination of both CAP and BV therapies or by the supplementation of each one alone. The combinatory effect was the most effective in suggesting correlated and synergistic mechanisms that influence their potent outcome when compared to other options.

Regarding the comparison with the reference drug, MTX, the effects of CAP + BV treatment of the arthritic rats on MDA, GSH, and SOD levels were more potent than the effects of MTX. However, when compared to either CAP or BV, MTX was more successful. Previous studies have demonstrated the antioxidant impact of MTX at low dosages [83, 84]. According to Luna‐López et al. [83], low‐dose MTX uses an Nrf2‐dependent mechanism to cause an antioxidant hormetic response in rat primary astrocytes. Otherwise, MTX can directly scavenge free radicals at low doses without requiring the Nrf2‐activated pathway, according to Clemens et al. [84]. It is relevant here to mention that high doses and long‐term use of MTX frequently induce tissue toxicity by suppressing antioxidant enzymes and increasing free radicals, leading to damage of healthy tissues (hepatotoxicity, nephrotoxicity, and intestinal injury) [14–16].

Histologically, the joint section analyses showed a perfect correlation with the overall biomarker results, which included oxidative stress, paw edema, and the ratio of proinflammatory to anti‐inflammatory immune pathways, which were examined in various animal groups with arthritis. In addition to deep subcutaneous granulomatous inflammatory reactions that reflect the presence of rheumatoid nodules, the most common extra‐articular symptom observed in 15%–20% of adult RA patients, the histological examination of the RA group revealed obvious pathological alterations of established arthritis, such as an uneven fluctuating articular surface and pannus formation [85]. Furthermore, the ankle of arthritic rats also showed signs of depleted lymphoid follicles, hyperplastic synovial membrane, connective tissue growth, enlarged cartilage cells, and visible invasion of inflammatory cells. Our research’s biomarker assays previously demonstrated this, and it is in line with other literature [86]. On the other side of the study, the MTX, BV, CAP, and CAP + BV treatments suppressed synovitis, immune cell infiltration, synovial hypertrophy (pannus formation), cartilage surface erosion, and joint damage, with a recommendation for combinatory CAP + BV therapy with a less inflamed and immune normal arthritic condition.

According to Figure 5, RA is a chronic autoimmune disease that causes autoantibody production, synovial tissue proliferation, pannus development, and ultimately, bone and cartilage loss, all of which can result in systemic problems and early death. Activation of CD4^+^ T cells, pathogenic B cells, macrophages, and dysregulation of cytokines, chemokines, and autoantibodies are all part of the aberrant autoimmune response that is the hallmark of RA. TNF‐α is one of these proinflammatory mediators that is essential to the pathophysiology of RA because it activates a number of downstream signaling pathways that lead to synovial inflammation. Because of their anti‐inflammatory and antioxidant qualities, treatment with MTX, CAP, BV, and the combination of CAP + BV showed improvements in the structural integrity of the ankle joint (Figure 5).

## 5. Conclusions

Both BV and CAP showed promise as natural medicinal agents with anti‐inflammatory and antiarthritic qualities in this investigation. The reduction of edema, suppression of RA and ACCP production, reduction of MDA levels, and inhibition of *TNF-α*, *IL-1*β, *iNOS*, *eNOS*, *MMP-1*, and *MMP-3* gene expression all supported the effectiveness of these drugs. Together with an improved antioxidant defense system demonstrated by raised GSH levels,*GR* expression, and SOD activity, histological examination also showed improvements in joint abnormalities. These results imply that combination therapy has potential as a natural immune modulator to treat RA. An important limitation of this study is to scrutinize the mechanistic actions of BV and CAP and their combination through assessing the effects on NF‐κB, Nrf2/Keap1, and other molecular signaling pathways. In addition, to identify and compare the effects on the articular cartilages of different bones in the ankle, separate fixation, decalcification, and staining of the articular cartilages, rather than processing the entire joints, are required. Another third limitation is the assessment of the treatments on BW, food intake, and liver, kidney, and heart functions to assess their safety in arthritic rats. Moreover, further clinical studies are required to assess the safety and effectiveness of treatments in RA patients.

## Author Contributions

**Ahmed Hussain**: investigation, data curation, formal analysis, visualization, writing – original draft, writing – review and editing. **Amira Sh. Soliman**: supervision, visualization, writing – review and editing. **Mohamed Abd-Elbaset:** investigation, data curation, visualization, writing – original draft, writing – review and editing. **Areej A. Al-Khalf:** funding acquisition, project administration, investigation visualization, writing – review and editing. **Eman A. Ahmed:** data curation, formal analysis, visualization, writing – original draft, writing – review and editing. **Abdelwahab Khalil:** investigation, data curation, formal analysis, visualization, writing – original draft, writing – review and editing. **Osama M. Ahmed:** supervision, investigation, data curation, formal analysis, visualization, writing – original draft, writing – review and editing. **Mohamed S. Abbas**: supervision, visualization, writing – original draft, writing – review and editing.

## Funding

This research was funded by the Princess Nourah bint Abdulrahman University researchers supporting project (Number PNURSP2026R37), the Princess Nourah bint Abdulrahman University, Riyadh, Saudi Arabia.

## Disclosure

All authors have read and approved the final version of the manuscript and agree to be accountable for all aspects of the work.

## Conflicts of Interest

The authors declare no conflicts of interest.

## Data Availability

The data that support the findings of this study are available from the corresponding author upon reasonable request.
